# Countering Calcium Blooming With Personalized Contrast Media Injection Protocols: The 1-2-3 Rule for Photon-Counting Detector CCTA

**DOI:** 10.1097/RLI.0000000000001078

**Published:** 2024-05-15

**Authors:** Michael C. McDermott, Thomas Sartoretti, Lion Stammen, Bibi Martens, Gregor Jost, Hubertus Pietsch, Ralf Gutjahr, Bernhard Schmidt, Thomas G. Flohr, Hatem Alkadhi, Joachim E. Wildberger

**Affiliations:** From the Department of Radiology and Nuclear Medicine, Maastricht University Medical Center, Maastricht, the Netherlands (M.C.M., T.S., L.S., B.M., T.G.F., J.E.W.); Diagnostic and Interventional Radiology, University Hospital Zurich, University of Zurich, Zurich, Switzerland (T.S., H.A.); Cardiovascular Research Institute Maastricht, Maastricht University, Maastricht, the Netherlands (M.C.M., T.S., L.S., B.M., J.E.W.); Bayer AG, Berlin, Germany (M.C.M., G.J., H.P.); and Computed Tomography Division, Siemens Healthineers AG, Forchheim, Germany (R.G., B.S., T.G.F.).

**Keywords:** CT, contrast media, injection protocol, CT angiography, photon-counting detector CT, iodine, calcium, blooming

## Abstract

**Objective:**

Photon-counting detector computed tomography (PCD-CT) enables spectral data acquisition of CT angiographies allowing for reconstruction of virtual monoenergetic images (VMIs) in routine practice. Specifically, it has potential to reduce the blooming artifacts associated with densely calcified plaques. However, calcium blooming and iodine attenuation are inversely affected by energy level (keV) of the VMIs, creating a challenge for contrast media (CM) injection protocol optimization. A pragmatic and simple rule for calcium-dependent CM injection protocols is investigated and proposed for VMI-based coronary CT angiography with PCD-CT.

**Materials and Methods:**

A physiological circulation phantom with coronary vessels including calcified lesions (maximum CT value >700 HU) with a 50% diameter stenosis was injected into at iodine delivery rates (IDRs) of 0.3, 0.5, 0.7, 1.0, 1.5, 2.0, 2.5, and 3.0 g I/s. Images were acquired using a first-generation dual-source PCD-CT and reconstructed at various VMI levels (between 45 and 190 keV). Iodine attenuation in the coronaries was measured at each IDR for each keV, and blooming artifacts from the calcified lesions were assessed including stenosis grading error (as % overestimation vs true lumen). The IDR to achieve 300 HU at each VMI level was then calculated and compared with stenosis grading accuracy to establish a general rule for CM injection protocols.

**Results:**

Plaque blooming artifacts and intraluminal iodine attenuation decreased with increasing keV. Fixed windowing (representing absolute worst case) resulted in stenosis overestimation from 77% ± 4% at 45 keV to 5% ± 2% at 190 keV, whereas optimized windowing resulted in overestimation from 29% ± 3% at 45 keV to 4% ± 1% at 190 keV. The required IDR to achieve 300 HU showed a strong linear correlation to VMI energy (*R*^2^ = 0.98). Comparison of this linear plot versus stenosis grading error and blooming artifact demonstrated that multipliers of 1, 2, and 3 times the reference IDR for theoretical clinical regimes of no, moderate, and severe calcification density, respectively, can be proposed as a general rule.

**Conclusions:**

This study provides a proof-of-concept in an anthropomorphic phantom for a simple pragmatic adaptation of CM injection protocols in coronary CT angiography with PCD-CT. The 1-2-3 rule demonstrates the potential for reducing the effects of calcium blooming artifacts on overall image quality.

The utilization of coronary computed tomography angiography (CCTA) is increasing due to its high sensitivity and negative predictive value, and has recently emerged as the primary noninvasive imaging modality for the diagnostic workup of low and medium risk patients with suspected coronary artery disease.^1^ There remain a few barriers to more widespread adoption of CCTA as the general primary imaging modality in the rule out of coronary artery disease across all risk categories.

Among these barriers is the possible degradation of image quality and subsequent diagnostic accuracy caused by blooming artifacts from coronary calcified lesions, which are more prevalent in the higher risk population.^2,3^ These blooming artifacts may obscure unobstructed vessel lumen and can lead to false-positives from overestimation of stenoses.^2^ Further, characterization of plaque composition in mixed-density lesions can be inhibited by these blooming or partial volume artifacts.^4^

Traditionally, CCTA images are acquired on an energy-integrating detector CT system with a single- or dual-source configuration in single-energy (SE) mode. In this mode, a single polychromatic image series is derived, with windowing as the primary mechanism to counteract the blooming artifacts. A step forward was made with the introduction of dual-energy (DE) CT, which enables virtual monoenergetic image (VMI) reconstructions at individual energy levels (eg, 40–190 keV) as compared with the traditional polychromatic images. Reconstructing images at higher keV enables an overall reduction in the blooming artifacts associated with calcified plaques and stents,^5^ albeit with a reduction in image contrast associated with iodine due to its absorption characteristics. Further, material decomposition is possible with the DE technique, which can allow for the separation of iodine, calcium, and other tissue based on differences in those same absorption characteristics.^6^ Due to technical constraints, such as limited temporal resolution or high radiation dose, DE CCTA has not entered standard clinical routine, and the majority of CCTA scans are still performed in SE mode.

A further advancement came with the recent clinical introduction of a whole-body dual-source photon-counting detector CT (PCD-CT) system equipped with cadmium telluride detectors enabling a direct conversion of detected x-ray photons to electrical signal. This allows for routine VMI reconstructions as well as spectral material decomposition, while maintaining high temporal resolution of dual-source imaging (necessary for cardiac CT examinations) and also improving spatial resolution, contrast-to-noise ratio, and lowering image noise as compared with energy-integrating detector CT systems.^7–14^ This is a benefit over other scanner technology capable of spectral acquisition (eg, dual-source/dual-energy, kV-switching, dual-layer, etc) as the corresponding reduced temporal resolution and/or increased radiation dose does not enable these technologies and scan modes to be widely used for CCTA specifically.

As the image acquisition technology has evolved, the opportunity has been taken to modify the injection protocols accordingly, usually with the intention to reduce the contrast media (CM) dose.^15,16^ Examples include the 10-to-10 rule, a simple clinical rule-of-thumb for SE acquisitions designed to adapt the CM injection protocol to the tube voltage selected. In this rule, with every 10 kV lower in tube voltage from a reference (eg, 120 kV), a corresponding 10% reduction in CM dose is enabled (as iodine attenuation increases at lower kV).^17^ Alternatively, with VMI reconstructions, visualization at lower energy levels (lower keV) enabled reduction of CM dose due to the increase in iodine attenuation as the keV levels approach the K-edge of iodine.^18,19^

Despite the substantial literature concerning CM dose reduction, little attention has been paid to the opportunity to further personalize CM injections to improve diagnostic image quality in challenging populations, like those with densely calcified lesions. The critical confounding variable that must be decoded is the relationship between calcium blooming artifacts and intravascular iodine attenuation. As aforementioned, blooming artifacts have been found to reduce as the corresponding intravascular attenuation of iodine exponentially decreases.^20^ The clinical implication is that as the ability to visualize the calcified lesion improves, the overall vessel lumen contrast provided by the iodine degrades. It is hypothesized, based on the physics of iodine attenuation under x-ray, that there exists a specific iodine delivery rate (IDR) to achieve a diagnostic threshold of 300 HU attenuation in the coronaries at each VMI energy level, and an equation describing the relationship may be established. This study aimed to establish that simple rule for optimization of CM injection protocols based on calcification density on coronary artery calcium scoring scans to reduce the effect of calcium blooming artifacts on diagnostic image quality while maintaining adequate vessel contrast.

## MATERIALS AND METHODS

### Phantom Setup

**FIGURE 1:**
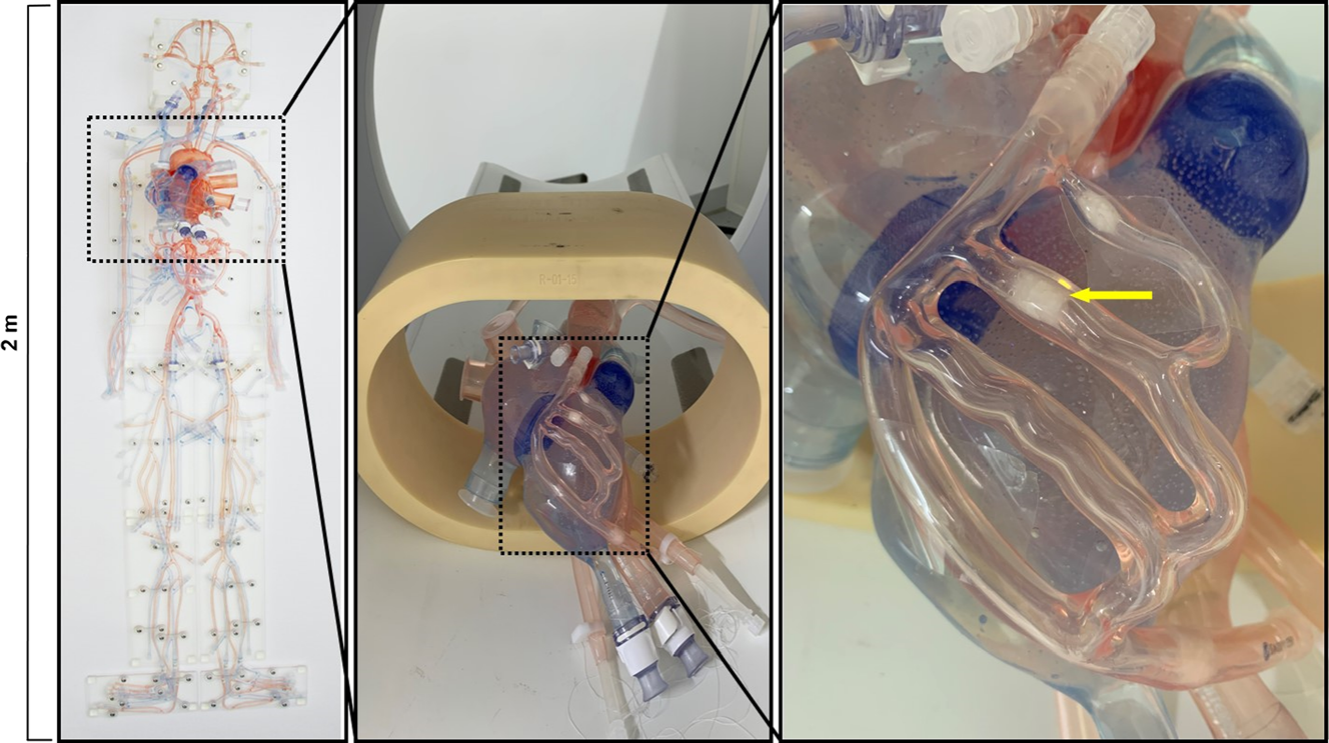
From left to right, left image of full body phantom, then middle image zoomed in on thorax and abdomen of the phantom showing heart model, then right is zoomed in image of the coronary vessels laid on top of the heart model including yellow arrows pointing to the calcified lesions within the coronary model. Images courtesy of United Biologics Inc, Santa Ana, CA.

The study was performed with a third-generation cardiovascular circulation phantom,^21^ including a physiological heart model and coronary branching vessels with simulated calcified lesions composed of deposited calcium carbonate (Fig. 1). The simulated calcified lesions were highly dense (maximum CT value >700 HU), heterogenous in shape, with maximum intralesion occlusion percentages (stenosis diameter) of 50% as defined by the phantom model vendor (United Biologics, Santa Ana, CA). In addition, an electromechanical pump (SuperPump; ViVitro Labs, Victoria, British Columbia, Canada) was connected to the phantom allowing for control over heart rate, stroke volume, and blood pressure, which together facilitate cardiac motion of the model and realistic contrast material transport and distribution dynamics. For image acquisition, the phantom was positioned inside an attenuating ring simulating thoracic tissue attenuation. The dynamic flow in the phantom was achieved with a setup consistent with previous literature,^22^ by circulating room temperature water at a heart rate of 60 beats per minute and a stroke volume of 80 mL to achieve a cardiac output of 4.8 L/min. Internal pressure of the phantom was maintained at 120/80 mm Hg.

### Contrast Media Injection Protocol

Injections were first performed with iopromide 370 (Ultravist; Bayer AG, Berlin, Germany) and a power injector (MEDRAD Centargo; Bayer AG, Berlin, Germany) into the phantom at IDRs of 0.3, 0.5, 0.7, 1.0, 1.5, 2.0, 2.5, and 3.0 g I/s. This range of IDRs was selected to cover the full expected clinical range of injected rates as well as the lower and upper extremes. The IDR was modified by maintaining the CM concentration (at 370 mg I/mL) and varying the flow rate between 0.81 mL/s and 8.1 mL/s. Injection volume was adjusted to maintain a constant injection duration of 10 seconds. A Coriolis flow meter (MicroMotion 5700; Emerson Electric Co, St Louis, MO) was used to measure the concentration of iodine entering the coronary vessels throughout the injection duration and the following 60 seconds. The peak iodine concentration was recorded and averaged across 5 repeated trials for each flow rate. For the subsequent CT scans, the coronaries were manually filled with solutions of iodinated CM titrated with deionized water to concentrations matching those calculated for the full set of given IDRs using the Coriolis meter. Although connected to the phantom, the coronaries were then isolated from the dynamic flow through the rest of the vasculature to ensure precise concentration of CM in the target vessels throughout the various trials and to protect the geometry of the coronary plaques.

### CT Image Acquisition

All scans were acquired on a first-generation, whole-body, dual-source PCD-CT system (NAEOTOM Alpha, Version VA50; Siemens Healthineers AG, Forchheim, Germany) equipped with 2 PCDs (cadmium telluride), each with a 144 × 0.4-mm collimation. First, electrocardiogram-triggered sequential acquisitions were performed in the spectral (QuantumPlus) mode. Tube voltage was set at 140 kVp to enable maximum spectral separation, a collimation of 144 × 0.4 mm was used, and the image quality (IQ) level was set to 60. Gantry rotation time was 0.25 seconds, resulting in a temporal resolution of 66 milliseconds. For all scans, radiation dose values were as follows: CTDI_vol_ of 3.03 ± 0.67 mGy and DLP of 53.3 ± 8.34 mGy·cm.

### CT Image Reconstruction

Virtual monoenergetic PCD-CT images were reconstructed from 45–95 keV in 10 keV increments, as well as at 100, 120, and 190 keV using a Bv56 kernel. Images were reconstructed with a slice thickness 0.4 mm and increment of 0.3 mm and quantum iterative reconstruction at strength level of 4. These settings are typical for CCTA with PCD-CT.^19^ A matrix size of 512 × 512 was used, and a field of view of 200 × 200 mm^2^ was selected as previously suggested.^5,23^ Although ultra-high resolution is possible with slice thickness of 0.2 mm and 1024 × 1024 matrix size, spectral information can so far only be exploited in standard scan mode at reduced spatial resolution on the clinical PCD-CT as compared with ultra-high resolution-CCTA. Therefore, the aforementioned parameters were selected.

### Data and Statistical Analysis

Two readers, including one board-certified radiologist with more than 5 years' experience and a second reader with more than 4 years' experience reading radiologic images, performed quantitative measurements by placing a circular region of interest in the coronary branching vessels for each image series combination at each keV level specified above. The mean vessel attenuation at each IDR was obtained across each measured keV level. Blooming artifacts associated with the calcified lesions were then quantified via measurements of the maximum linear external plaque diameter and in-plane internal lumen diameter on long axis views using manual double-oblique reconstructions. Percentage blooming artifacts were approximated with the formula below:^5^


$$\text{Blooming artifact}\left(\%\right)=\begin{array}{l} \frac{\text{External calcified plaque diameter}-\text{Lumen diameter}}{\text{External calcified plaque diameter}}\\ \times 100 \end{array}$$

A stenosis grade was then recorded as an expected percentage occlusion by the reader, with the percent error calculated using the formula below:


$$\text{Stenosis grade error}\;\left(\%\right)=1-\frac{\text{Measured lumen diameter}}{\text{True lumen diameter}}\times 100$$

All measurements were first performed with a fixed window/level-setting at 1100/400 C/W for all keV levels, selected to enable minimum visualization at 45 keV (images overly bright with 800/200 default window/level-setting from the vendor). Although fixed windowing may not reflect clinical practice, it was included to represent an upper bound for the error as an absolute worst-case scenario, without the counteracting effect of reducing calcium blooming through optimized windowing. Measurements were then repeated with the reader allowed unlimited time to adjust the windowing for optimized visualization, representing a “best-case scenario” as unlimited time is not typically afforded in routine clinical practice. The inclusion of both data points enables a representative error region/band for the evaluation of this particular lesion, with the true error likely falling somewhere in between depending on reader experience and W/L optimization. The inclusion of both data points enables a representative error region/band for the evaluation of this particular lesion, with the true error likely falling somewhere in between depending on reader experience and available time for W/L optimization. The blooming artifacts and stenosis grading errors were then evaluated versus keV level to determine a range appropriate for visualization of densely calcified lesions. All analyses were performed using R statistical software (R Core Team, version 4.1.1; R Foundation for Statistical Computing, Vienna, Austria). Data are reported as mean ± SD, and correlation coefficients are calculated using Spearman method with a standard Grubb test performed to evaluate for outliers.

### Deriving a Rule of Thumb

A diagnostic threshold of 300 HU in the coronaries was used to goal seek a corresponding injected IDR needed to achieve that HU threshold at each keV level.^5,24,25^ The necessary IDRs obtained in this step were then compared with the appropriate keV levels from the blooming artifact assessment to deduce a general rule for calcium-dependent contrast injection protocols.

## RESULTS

As part of the initial setup and validation process, injected IDR demonstrated strong linear correlation to the measured iodine concentration in the coronary arteries using the Coriolis meter (*R*^2^ = 0.997). The concentrations of the titrated solutions prepared for use in the isolated coronary vessels were measured and confirmed to be within 1 standard deviation of the real concentration measurements in the dynamic vessel phantom, as shown in Table 1. Correlation to corresponding attenuation at 120 kVp (using conversion factor of 25 HU/mg I/mL) yields exemplary values of 406 HU with an IDR of 2 g I/s, and 307 HU with an IDR of 1.5 g I/s.

**TABLE 1:** Correlation of IDR to Iodine Concentration in the Coronary Arteries and Corresponding Vessel Contrast at Reference 120 kVp SE Acquisition

Iodine Delivery Rate (g I/s)	Iodine Concentration in Coronaries (mg I/mL)	Measured Titration Concentration (mg I/mL)	Calculated Attenuation With Reference 120 kVp SE Acquisition
3.0	24.17 ± 0.14	24.23	604
2.5	20.20 ± 0.12	20.30	505
2.0	16.24 ± 0.11	16.34	406
1.5	12.27 ± 0.09	12.18	307
1.0	8.31 ± 0.05	8.33	208
0.7	6.33 ± 0.06	6.35	158
0.5	4.34 ± 0.09	4.34	109
0.3	2.76 ± 0.13	2.76	69

**FIGURE 2:**
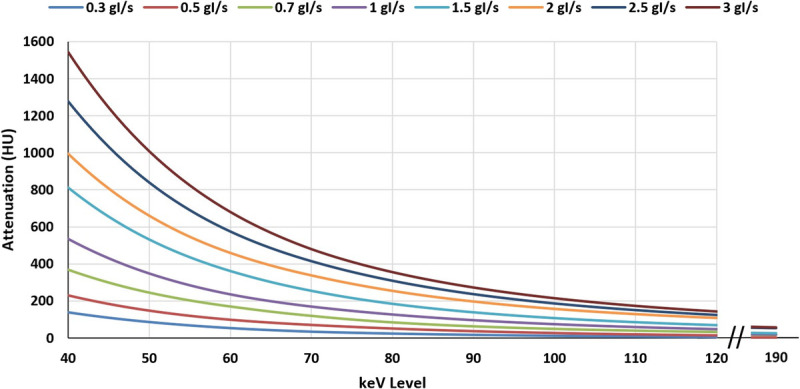
Vessel contrast as measured in the coronary branching vessels versus injected iodine delivery rate at varying keV levels.

**FIGURE 3:**
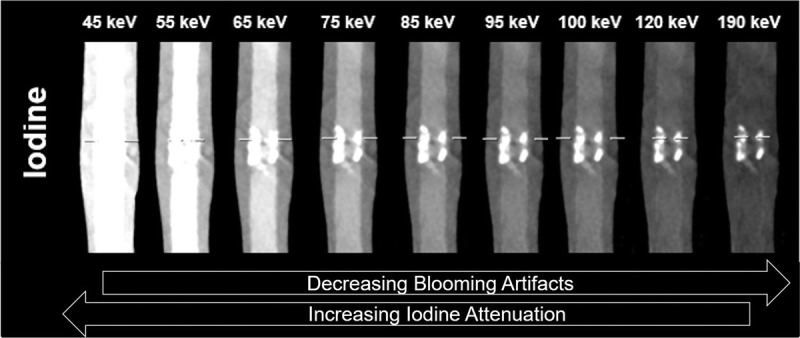
Visualization with vendor-default, nonoptimized window setting of 800/200 of same coronary calcified lesion at increasing keV levels from left to right including vessel contrast generated by an injection at an IDR of 1.5 g I/s. White dashed lines represent the plane of maximum occlusion.

**FIGURE 4:**
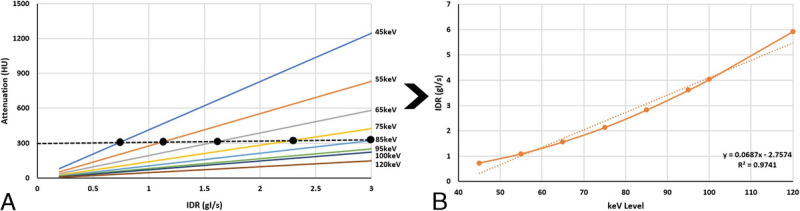
A, Graphical representation of goal-seek calculation to acquire the necessary IDR at each keV level to achieve a threshold of 300 HU. B, Plot of the calculated IDRs from panel A necessary to be injected to achieve a vessel attenuation of 300 HU at varying keV levels.

The intraluminal attenuation in the coronary vessels demonstrated perfect linear correlation (*R*^2^ = 1) with increasing attenuation as IDR was increased. Similarly, an increase in attenuation was observed at each IDR with decreasing keV level. The results are displayed in Figure 2. A maximum (of the mean attenuation measurements) of 1033 HU was achieved at 45 keV with an IDR of 3 g I/s, whereas a minimum of 0.88 HU was achieved at 190 keV with an IDR of 0.3 g I/s. The qualitative blooming artifacts associated with the calcified lesion decreased with increasing keV level, as visualized in Figure 3. When solving for the IDR required to achieve 300 HU in the coronaries at each keV level, as visualized in Figure 4A, a strong linear correlation was achieved (*R*^2^ = 0.98) as shown in Figure 4B.

**FIGURE 5:**
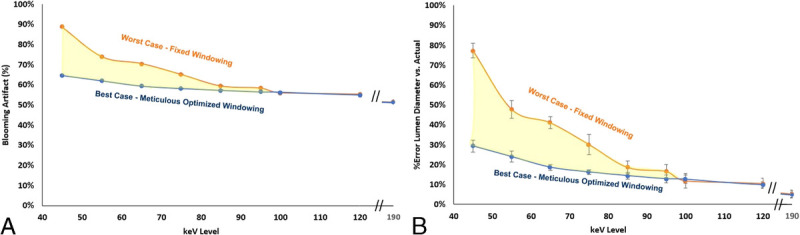
A, Plot of calculated blooming artifact versus keV level for worst-case fixed windowing scenario and best-case time-unbounded optimized windowing with shaded region representing area within which blooming artifact percentage is likely to be expected for this lesion. B, Plot of the % overestimation of the stenosis by the readers under the 2 scenarios of fixed windowing and time-unbounded optimized windowing, with shaded region representing area within which stenosis overestimation % can be likely expected for this lesion.

For quantitative evaluation of blooming artifacts, with fixed window settings of 1100/400, the maximum plaque blooming artifact was calculated as 89% ± 0.3% at 45 keV, whereas the minimum plaque blooming artifact was calculated as 52% ± 0.2% at 190 keV. With time-unbounded optimized windowing, the maximum plaque blooming artifact was calculated as 65% ± 0.2% at 45 keV, whereas the minimum plaque blooming artifact was calculated as 52% ± 0.2% at 190 keV. The complete results for blooming artifacts are shown in Figure 5A.

The stenosis grading error represented as % overestimation for the fixed windowing was recorded with a maximum of +77% ± 4% at 45 keV (reported as average of 2 readers ± standard deviation) and a minimum of +5% ± 2% at 190 keV. With time-unbounded optimized windowing, the maximum overestimation was recorded as +29% ± 3% at 45 keV, and a minimum of +4% ± 1% at 190 keV. The complete results for stenosis grading error are shown in Figure 5B.

## DISCUSSION

In this study using a third-generation physiological cardiac motion phantom for CCTA with dual-source PCD-CT, we found a strong correlation between keV level and calcium blooming artifacts, with results consistent with those previously reported in literature.^20^ In addition, a strong correlation was found between keV level and intravascular iodine attenuation at different IDRs. As expected, the intraluminal attenuation was found to increase with increasing IDR and also with decreasing keV level. This relationship is due to the spectral properties of the iodinated CM, as aforementioned, the attenuation of the iodinated CM decreases exponentially with increased keV.^21^ A CM with optimized attenuation properties would have a horizontal line of attenuation when plotted versus keV level, thus providing consistent attenuation regardless of the acquisition or reconstruction parameters. Contrast agents from metals with high atomic number (eg, tungsten-based) may represent a more optimal CM in this regard.^6^

Qualitatively, the plaque blooming artifacts increased with decreasing keV level, and this was further backed up with quantified plaque blooming artifact percentages calculated at both a fixed window/level setting (representing a worst-case scenario) and a time-unbounded optimized window/level setting (representing a best-case scenario). It should be noted that this calculation for plaque blooming artifact may be less relevant for use in a phantom where the silicone wall vessel thickness is much greater than would be expected in vivo. This is evidenced by approximately zero standard deviation between the 2 readers in % despite nonzero differences in the absolute value of their inner lumen diameter measurements. However, the trend across keV levels remains valid, and as there is a desire clinically to decrease the artifacts associated with calcified plaque blooming while ensuring sufficient vessel contrast for diagnostic assessment, these 2 relationships are suboptimal.

The stenosis overestimation as measured by 2 independent readers in this study reflects the clinical significance of these findings, as the overestimation for this particular lesion volume, density, and complexity at 45 keV was found to be between 29% and 77%. This has the potential in a patient population to result in an inaccurate representation of the significance of the stenosis and potentially unnecessary downstream testing such as catheter angiography.

Based on the results of this study, for patients with nonzero coronary calcium burden, the reader should expect to visualize VMIs at higher energy levels to minimize the impacts of the plaque blooming artifacts, especially those associated with very dense and complex calcifications. However, as shown here, to maintain the vessel contrast from the iodine above a diagnostic threshold, the injected IDR must be adapted. Critically, at the point of reporting, the examination is unfortunately already completed and there is no longer an opportunity to increase the amount of iodine to compensate for the need of the reader to visualize the calcifications at higher keV levels. Therefore, an adaptation must be made during the time of image acquisition. One could theoretically increase the total iodine load and IDR to every patient to ensure there is sufficient iodine present in those patients with severely calcified lesions; however, this leads to potentially harmful and unnecessary additional CM exposure to many patients.

**FIGURE 6:**
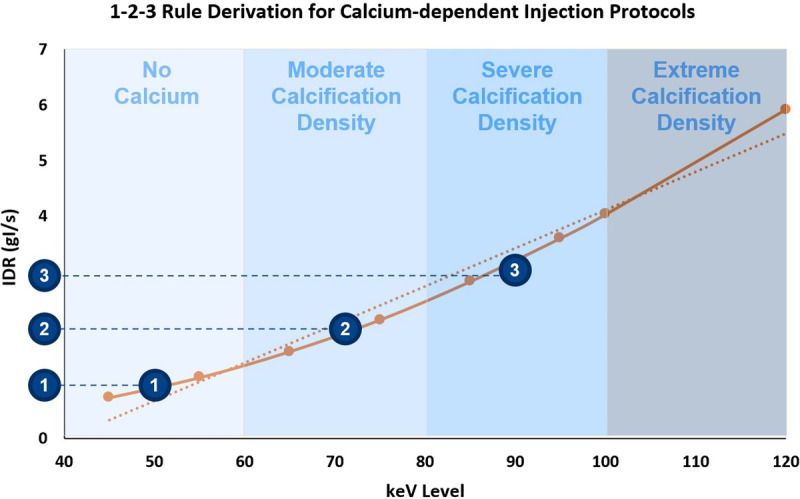
Depiction of the derivation of the 1-2-3 rule with plot of IDRs necessary to achieve 300 HU in the coronary arteries across varying energy levels with an overlay of generalized calcification density along a spectrum. Respective IDRs needed to meet the diagnostic 300 HU threshold at the center point of each bucket.

A proposed solution to this problem is to evaluate the calcium burden and calcification densities of the patient as part of the coronary calcium scoring acquisition (typically performed prior to CTA), and updating the injection protocol based on the calcification density accordingly. Figure 6 represents the same graph displayed in Figure 4B; however, highlighting 4 different potential regimes that can be considered in clinical practice.

For patients with no calcium burden, visualization of VMIs at 40–60 keV is reasonable and consistent with the literature.^22–26^ For moderate calcification density, it is likely that visualization at higher keV levels is more appropriate (eg, 60–80 keV). Patients with severely dense calcifications would likely require still higher keV levels to minimize the blooming artifacts (eg, 80–100 keV). For extremely dense calcifications, energy levels greater than 100 keV are likely required.^27–30^ However, according to the results from Figure 4B, energy levels beyond 90 keV would require IDRs that approach and exceed the limits of power injectors to deliver iodine at higher rates (IDR of 3 g I/s corresponds to between 7.5 and 10 mL/s depending upon the iodine concentration of the CM used, which is typically between 300 and 400 mg I/mL). When taking the center point of these 4 proposed regions and evaluating the corresponding IDRs from the y-axis, it can be seen that patients with calcifications of a moderate density would require twice the IDR of a patient with no calcium burden, whereas patients with severely dense calcifications would require 3 times the IDR.

**FIGURE 7:**
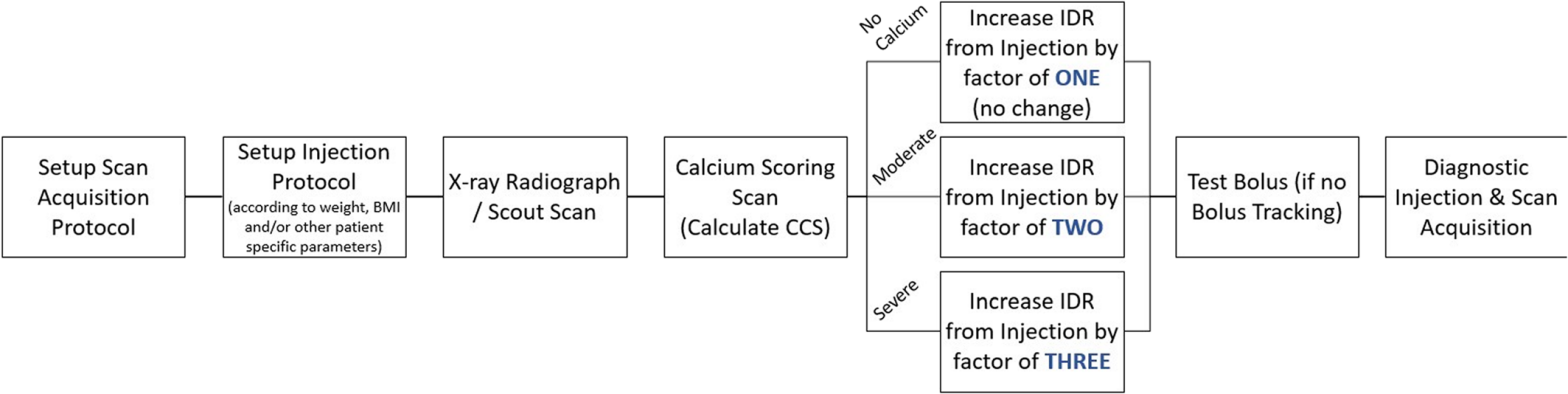
Schematic flow diagram of the 1-2-3 rule implementation proposal in clinical routine for cardiac CT examinations.

Based on these results, a simple rule can be created, hereafter referred to as the 1-2-3 rule. The schematics of the 1-2-3 rule are shown in Figure 7. In essence, once a reference protocol is established for a given patient (eg, based on body weight and other well-known factors^31^), the calcium scoring scan is conducted. If there is no calcium present, the reference protocol stays unchanged (multiplied by a factor of 1). If there are calcifications with moderate to low density observed, the reference injection protocol should be multiplied by a factor of 2. If there are calcifications observed with severe density, the reference injection protocol should be multiplied by a factor of 3. In all cases, the injection volume should be correspondingly adapted to maintain a desired injection duration appropriate for the scan acquisition type (eg, prospectively triggered sequential, high-pitch spiral, etc). It should be noted that IDRs of 3.0 g I/s (which could translate to flow rates of up to 10 mL/s for 300 mg I/mL contrast agents) result in somewhat extreme flows for peripheral venous power injection and likely are not able to be achieved in clinical practice. This may inherently limit the ability to optimize the image quality for patients with severely calcified plaques of extreme volume, density, and/or complexity. It is for these reasons that a spectrally optimized contrast agent would ideally be developed, which would allow for sufficient iodine attenuation at higher energy levels without needing to significantly increase IDR to extreme levels.

Despite the upper end limitations, the use of this pragmatic rule in clinical routine could potentially bring benefits with further personalization of the CM injection protocol and a reduction of plaque blooming artifacts in CCTA that can result in unnecessary downstream tests. However, there is further investigation in a patient population that is needed to validate the results, as well as to provide more certainty on the thresholds among the 4 buckets of calcification severity this rule has defined (perhaps based on coronary calcium score value).

A significant limitation of this study is that it was conducted only in an ex vivo representative phantom with a single representation of calcification density and complexity. Although calcium volume, density, and distribution will of course affect the absolute magnitude of the values displayed in Figure 5, and to some degree the optimal keV level for visualization, this does not change the applicability of the 1-2-3 rule, which enables sufficient iodine attenuation in the vessel lumen across the keV levels, and is independent of the characteristics of the plaque. Regarding the use of a phantom, systematic evaluation of varying CM injection and scan parameters as conducted herein is not otherwise possible in a human population for ethical reasons. It is expected that variations in body habitus, cardiovascular function (eg, cardiac output and heart rate variability), and pathophysiology (including varying distribution characteristics of the calcium within the vessel lumen) will differ significantly between patients, causing variability in the absolute magnitude of intraluminal attenuation at a given IDR and the appropriate keV level for optimal visualization. However, this study suggests only a scalar multiplier of the IDR from a reference protocol that is set by the respective clinics based on their existing protocols. Any additional confounding variables not yet accounted for (eg, heart rate variability) will be included in the existing empirically derived reference protocol. This 1-2-3 rule is agnostic of the absolute value of attenuation but focused only on the relative modifications within a given patient as the coronary calcification density and complexity falls along a spectrum. Consistent with previous efforts to optimize CM injection protocols, this result should anyways be followed up with a prospective patient study.

This study provides a proof-of-concept in an anthropomorphic phantom for a simple pragmatic adaptation of CM injection protocols in CCTA with PCD-CT. The 1-2-3 rule demonstrates the potential for reducing the effects of calcium blooming artifacts on overall image quality. In addition to validation in prospective patient studies as a next step, evaluation of more spectrally optimized CM for use in these applications to replace iodine should be further explored.
